# Complement C3 Enhances LPS-Elicited Neuroinflammation and Neurodegeneration Via the Mac1/NOX2 Pathway

**DOI:** 10.1007/s12035-023-03393-w

**Published:** 2023-06-03

**Authors:** Ran Zhou, Shih-Heng Chen, Zhan Zhao, Dezhen Tu, Sheng Song, Yubao Wang, Qingshan Wang, Jing Feng, Jau-Shyong Hong

**Affiliations:** 1 Respiratory Department, The First People’s Hospital of Yunnan Provience, Kunming, 650032 People’s Republic of China; 2 Neurobiology Laboratory, National Institute of Environmental Health Sciences, National Institutes of Health, 111 T.W. Alexander Dr., Research Triangle Park, Durham, NC 27709 USA; 3 Respiratory Department, Tianjin Medical University General Hospital, Tianjin, 300052 People’s Republic of China; 4 Department of Neurology, University of North Carolina, Chapel Hill, NC 27599 USA; 5 School of Public Health, Dalian Medical University, Dalian, Liaoning Province China

**Keywords:** Complement C3, Mac1 receptor, NADPH Oxidase (NOX2), Microglia, Astroglia, Neuroinflammation, Neurodegeneration

## Abstract

Recent studies showed increased expression of complements in various neurodegenerative diseases, including Alzheimer’s and Parkinson’s diseases. However, the mechanism regulating the expression of complements and their roles in the pathogenesis of neurodegeneration are unclear. We hypothesized that acute neuroinflammation increases the expression and activation of brain complements, which, in turn, participate in chronic neuroinflammation and progressive neurodegeneration. We initially focused on the complement component C3, because C3 can activate microglia by binding to C3 receptors and attaching to damaged neurons destined to be phagocytosed by microglia. We found that complement C3 is upregulated in lipopolysaccharide (LPS)-stimulated neuron/glial cultures. Mechanistic studies revealed that microglia-released proinflammatory factors initiated the enhanced expression of C3 in astroglia during acute neuroinflammation. On the other hand, the sustained C3 expression during chronic neuroinflammation requires releasing damage-associated molecule patterns (DAMPs) from damaged/degenerating brain cells. Our results suggested that DAMPs might act on microglial integrin receptor Mac1 to trigger the activation of NADPH oxidase (NOX2). Activated microglial NOX2 increases the production of extracellular reactive oxygen species (ROS), elevating the levels of intracellular ROS of astroglia and sustaining the astroglial C3 expression. This was supported by the findings showing reduced C3 expression and attenuated neurodegeneration in LPS-treated neuron/glial cultures prepared from mice deficient in Mac1 or NOX2. LPS-induced neurodegeneration and oxidative stress are significantly reduced in C3 KO neuron/glial cultures and mouse brains. Together, this study provides the first evidence demonstrating the role of C3 in regulating chronic neuroinflammation and in driving progressive neurodegeneration.

## Introduction

The complement system plays a critical role in innate immunity, which helps to recognize, track, and eliminate exogenous and endogenous danger signals from pathogens or damaged/dead cells, such as pathogen-associated molecular patterns (PAMPs) and damage-associated molecular patterns (DAMPs), respectively [1]. The complement system consists of more than 30 fluid-phase and cell-associated proteins, and the liver is the primary source of complements in systemic circulation [2, 3]. Peripheral complements cannot gain access to the brain unless the blood–brain barrier (BBB) is damaged or compromised. Therefore, the complements in the brain are synthesized locally by glial cells and neurons. During neuroinflammation, the complements can be activated through classical, lectin, or alternative pathways by different types of danger signals, which help the phagocytosis of pathogens and unwanted host materials, enhance cytokine production, and form membrane attack complex (MAC) to lyse the bacteria or infected host cells [4]. Complement C3 is the center of the complement cascade; it is the point of convergence for all three main activation pathways. All three complement activation pathways lead to the assembly of C3 convertases, which, in turn, can cleave C3 into C3a and C3b, which play critical roles in immune activation and neuroinflammation [5]. C3a is an anaphylatoxin that can increase vasodilation via smooth muscle contraction, vascular permeability, and the release of histamine from innate immune cells. C3b is an opsonin that facilitates the phagocytosis of pathogens, cell debris, or mutant proteins by immune cells (e.g., microglia) expressing its receptor Mac1 (also knowns as complement receptor 3 [CR3], CD11b/CD18, or α_M_β_2_) [6-9]. Accumulating evidence shows that complement C3 is present in patients of all major neurodegenerative diseases, including Parkinson’s disease, Alzheimer’s disease, Huntington’s disease, and amyotrophic lateral sclerosis [6-9]. However, the function of C3 and how it is regulated during neuroinflammation, and its role in subsequent neurodegeneration are still not fully understood.

Previous studies from our group and others have demonstrated the C3 receptor (Mac1) is essential in maintaining chronic reactive microgliosis and in driving inflammation-mediated neurodegeneration [10, 11]. Additionally, the expression of Mac1 is increased in the brains of patients with Alzheimer’s disease and lipopolysaccharide (LPS) and MPTP animal models of Parkinson’s disease [12, 13]. Besides binding to C3, microglial Mac1 is also capable of binding DAMPs released from damaged neurons, including α-synuclein, β-amyloid, HMGB-1, and myelin, to initiate and maintain reactive microgliosis and triggers chronic neuroinflammation and subsequent neurodegeneration [14]. Recent studies have indicated that microglial NADPH oxidase (NOX2) is an important downstream effector of Mac1 signaling [14]. Upon Mac1 stimulation, NOX2 cytosolic subunits translocate and bind to membrane subunits to assemble the active form of NOX2 that generates superoxide and reactive oxygen species (ROS) metabolites. ROS enhances the oxidative stress in neurons that impairs neuronal mitochondrial functions and results in their degeneration [15-17]. In addition, ROS also act as secondary messengers responsible for regulating proinflammatory gene expression in the brain that causes collateral damage to neighboring neurons [18-20].

The purposes of this study were to understand the mechanism underlying C3 expression and its function in chronic neuroinflammation and progressive neurodegeneration. We used LPS to induce neuroinflammation in both in vitro and in vivo models using wild-type, C3-deficient, Mac1-deficient, and NOX2-deficient mice. The results showed that the Mac1-NOX2 signal pathway is critical in regulating C3 expression during chronic neuroinflammation. C3 expression was reduced in Mac1- and NOX2-deficient neuron-glial cultures. Like other DAMPs released from damaged neurons, C3 from activated astroglia participates in the feed-forward loop of reactive microgliosis, enhances the elevation of oxidative stress in neurons, and facilitates neuronal degeneration. The in vivo studies showed that LPS-induced oxidative stress was significantly reduced in complement C3-deficient mice. This study demonstrates that C3 plays a critical role in initiating, maintaining chronic neuroinflammation and triggering neurodegeneration.

## Materials and Methods

### Animal Treatment

Male C57BL/6 J, complement C3–deficient (C3KO, B6.C3-Tg [B6;129S4-*C3*^*tm1Crr*^/J), Mac1 = deficient (Mac1 KO, B6.129S4-Itgam < tm1Myd > /J), and NADPH oxidase–deficient (CYBB, B6.129S-Cybb < tm1Din > /J) mice were obtained from The Jackson Laboratory (Bar Harbor, ME). Housing and breeding of animals were performed humanely following the National Institutes of Health’s Guide for the Care and Use of Laboratory Animals (Institute of Laboratory Animal Resources, 2011). A single systemic injection of LPS (15 × 10^6^ EU/kg, i.p., *Escherichia coli* 0111: B4, Sigma-Aldrich, St. Louis, MO) was administered to 8–12-week-old C57BL/6 J and C3KO mice (B6.C3-Tg [B6;129S4-*C3*^*tm1Crr*^/J, The Jackson Laboratory) mice. Mice used as vehicle control were injected with saline (5 ml/kg, i.p.). Mice were sacrificed at different time points, and the brain tissues were collected for further analysis. In Fig. 5 D, mice were subcutaneously injected with saline or NADPH oxidase inhibitor, diphenyleneiodonium (DPI; 10 ng/kg), three times a day for 4 days before LPS administration and 6 and 12 h after LPS injection. Mice were sacrificed 24 h after LPS treatment to assay brain C3 mRNA. The dose of LPS and DPI was selected based on our previous study [21]. All mice were housed with 12 h of artificial light–dark cycle and provided fresh deionized or acidified water.

### Reagents

Poly-d-lysine, cytosine β-d-arabinofuranoside (Ara-c), l-leucine methyl ester (LME) and 3,3′-diaminobenzidine, mazindol, and urea-hydrogen peroxide tablets were purchased from Sigma-Aldrich (St. Louis, MO, USA). LPS (*E. coli* strain O111:B4) and NADPH oxidase inhibitor DPI were purchased from Sigma (San Diego, CA, USA). Cell culture ingredients were obtained from Life Technologies (Grand Island, NY, USA). Antibody diluents were purchased from DAKO (Carpinteria, CA, USA). Anti-complement C3 antibody, Complement C3, and iC3b protein (endotoxin-free) were purchased from Millipore Sigma, and the anti-Iba1 antibody was purchased from Wako Pure Chemicals (Richmond, VA, USA). Anti-3-nitrotyrosine (3-NT) and anti-tyrosine hydroxylase (TH) antibodies were purchased from Abcam (Cambridge, MA, USA). TNF-α ELISA kit and isotype control antibodies were purchased from R&D Systems (Minneapolis, MN, USA). Alexa Fluor 594 donkey anti-rabbit IgG and Alexa Fluor 488 donkey anti-goat IgG were purchased from cell signaling (Burlingame, CA, USA).

### Mesencephalic Neuron-glia Culture

Mouse mesencephalic neuron/glia cultures were prepared from C57BL/6 J, Mac1 KO, CYBB, or C3 KO mice as described previously [22-25]. Briefly, midbrain tissues were dissected from day 14 embryos and then gently triturated into a single-cell suspension. Cells were centrifuged and resuspended in warm MEM medium supplemented with 10% heat-inactivated fetal bovine serum (FBS) and 10% heat-inactivated horse serum, 1 g/L glucose, 2 mM l-glutamine, 1 mM sodium pyruvate, and 0.1 mM nonessential amino acids. Cells were then seeded (5.5 × 10^5^ cells/well) in poly-d-lysine (20 μg/ml) pre-coated 24-well plates. Cell counting was performed to ensure the equal number of cells was seeded when we used different types of cultures. The cultures were incubated at 37 C in 5% CO_2_ for 3 days and then replenished with 500 μl of fresh maintenance media. Cultures were treated 7 days after seeding. Immunocytochemistry revealed neuron/glial cultures contained ~ 50% astroglia (GFAP-immunoreactive cells), 40% neuron (MAP-2 immunoreactive cells), and 10% microglia (Iba-1 immunoreactive cells).

### Mesencephalic Neuron-Enriched Culture

Mesencephalic neuron/glia cultures from C57BL/6 J mice were prepared as described above. At 48 h after seeding, 20 µM cytosine β-d-arabinofuranoside (Ara-c) was added into the cultures to delete glial cells. After 3 days, media containing Ara-C was removed and replaced with fresh media. Neuron‐enriched cultures were ~ 98% pure.

### Primary Mixed Glial, Highly Enriched Astroglia, Highly Enriched Microglia, and Reconstitute Neuron-Microglial Cultures

Primary mixed glial cultures were prepared from C57BL/6 J mice at postnatal days 1–3, as previously described [26]. Briefly, cortices and midbrain were isolated and triturated into a single-cell suspension. Cells were centrifuged and resuspended in warm mixed glial culture medium (DMEM-F12 MEM supplemented with 10% heat-inactivated FBS and 2 mM l-glutamine, 1 mM sodium pyruvate, and 0.1 mM nonessential amino acids), and plated on poly-d-lysine pre-coated 6-well plates at 1 × 10^6^ cells/well. The medium was refreshed every 3 days until 14 days after seeding. Highly enriched astroglial cultures were derived from mixed glial cultures supplemented with 1 mM of LME at 72 h after seeding and cultured for 5–7 days. Immunocytochemistry revealed mixed glial cultures contained 15% microglia (Iba-1-immunoreactive cells), 3% oligodendrocytes (MBP-immunoreactive cells), and 80% astroglia (GFAP-immunoreactive cells), whereas highly enriched astroglial cultures contained less than 0.005% microglia, and more than 99.9% astroglia.

Microglia-enriched cultures were prepared from C57BL/6 J mixed glial cultures as described above. Microglia were isolated by shaking the flasks containing confluent mixed-glia cultures for 1.5 h at 180 rpm and replated on poly‐d‐lysine‐coated 24‐well plates at 5 × 10^5^ cells/well. Immunocytochemistry revealed that microglia‐enriched cultures contained less than 1% contamination of astroglia (GFAP‐immunoreactive cells).

For reconstitute neuron-microglia cultures, neuron-enriched cultures were prepared as described above, and the enriched microglia were directly plated on top of the existing neuron-enriched cultures to a ratio of 90% neurons and 10% microglia and treated 24 h later.

### Preparation of Glia-Conditioned Medium and Collection of DAMPs from the Supernatants of Neuron/Glia Cultures

#### Glia-Conditioned Medium

C57BL/6 J mixed glial cultures (contained ~ 15% microglia (Iba-1-immunoreactive cells), ~ 3% oligodendrocytes (MBP-immunoreactive cells), and ~ 80% astroglia (GFAP-immunoreactive cells) [24] were treated by 20 ng/ml LPS or control treatment medium, and then supernatants were collected 24 h after treatment.

#### Collection of DAMPs

C57BL/6 J neuron/glia cultures were treated by 20 ng/ml LPS or control treatment medium followed by warm saline washout 24 h after treatment to remove LPS and proinflammatory cytokines released from microglia and replenished with fresh medium. The DAMPs-containing medium was collected on day 5 after washed out.

### Immunocytochemical and Immunofluorescence Staining

For immunocytochemical staining, neuron glial cultures were fixed with 3.7% formaldehyde in PBS for 20 min. Fixed cultures were treated for 10 min with 1% hydrogen peroxide and incubated for 20 min in blocking solution (BSA 1%/Triton X‐100 0.4%/Normal Goat Serum 4% in PBS) to prevent nonspecific binding. Cells were immunostained overnight at 4 °C with rabbit polyclonal antibody against TH (1:5000; DA dopaminergic neuron marker), goat polyclonal antibody against C3 (1:6000), or mouse polyclonal antibody against 3-NT (1:4000) in Antibody Diluent (DAKO). Antibodies were detected with biotinylated secondary antibodies, i.e., goat anti-rabbit, horse anti-goat, or goat anti-mouse (1:227; Vector Laboratory), diluted in PBS containing 0.3% Tritone X-100 for 2 h. After washing three times with PBS, the cultures were incubated for 1 h with the Vectastain ABC reagents (Vector Laboratory, Burlingame, CA) diluted in PBS containing 0.3% Triton X-100. To visualize the signal, the cultures were incubated with 3,3′-diaminobenzidine and urea-hydrogen peroxide tablets dissolved in water. TH-positive cells were manually counted under a microscope (Nikon, model DIAPHOT, Garden City, NY, USA) by at least two investigators, and the results were averaged. The quantification of C3 and 3-NT was performed by ImageJ software. Briefly, the image was first converted into a grayscale picture, and the background was adjusted before the quantifying area was selected for the measurement of the total pixels. The relative density of the staining was compared based on the density of the total pixels of a certain region (total pixels/area).

For immunofluorescence staining, LPS- or vehicle-injected mice were perfused with saline followed by 4% paraformaldehyde (PFA), brains were removed and fixed in 4% PFA for 48 h, dehydrated with 30% sucrose, and then cut to brain slices with the thickness of 35 µm. The brain slices were placed in the blocking solution for 20 min (BSA 1%/Triton X-100 0.4%/normal goat serum 4% in PBS) to prevent nonspecific binding. Brain slices were immunostained overnight at 4 °C with mouse polyclonal antibody against 3-NT (1:1000) diluted in Antibody Diluent. Antibodies were detected and visualized using Alexa Fluor 488 goat anti-mouse IgG (1:1500) secondary antibodies. The images were acquired using a confocal laser-scanning microscope Zeiss 780. 3-NT fluorescent intensity was measured by ImageJ software.

### RNA Analysis

Total RNA was extracted from cultures and rodent brains with Qiagen RNeasy Minikit and reverse transcribed with an oligo dT primer. Real-time PCR amplification was performed using SYBR Green PCR Master Mix and a QuantStudio 6 Flex (Applied Biosystems, Therm Fisher Scientific) according to the manufacturer’s protocol. The primers were designed using Vector NTI software (v.11, Invitrogen, Carlsbad, CA) and validated for efficacy through melting curve analyses. Mouse C3 F (5′ CCA TC CAGC AGG TCA TCA AGT CAG 3′), mouse C3 R (5′ GCT GAT GAA CTT GCG TTG CTG C 3′), mouse GAPDH F (5′ TTC AAC GGC ACA GTC AAG GC 3′), and mouse GAPDH R (5′ GAC TCC ACG ACA TAC TCA GCA CC 3′) were used to amplify the mouse complement C3 gene. Amplifications were done at 95 °C for 10 s, 55 °C for 30 s, and 72 °C for 30 s for 40 cycles. All samples were tested in triplicate from at least three independent experiments and normalized with GAPDH using the 2^−∆∆Ct^ method.

### Western Blot

The protein extracts from cultured cells were homogenized in radioimmunoprecipitation assay lysis buffer (50 mM Tris–HCl, pH 8.0, 150 mM NaCl, 5 mM EDTA, 1% NP-40, 0.5% sodium deoxycholate, 0.1% SDS, and 1:100 protease inhibitor cocktail). Protein concentrations were determined using the bicinchoninic acid assay (Pierce) and denatured in the protein loading buffer. Equal amounts of protein were resolved on 4–12% Bolt^Tm^ Bis–Tris plus gel, and immunoblot analyses were performed using antibodies against C3 (1:1000; Millipore). An antibody against β-actin (1:5000; Cell Signaling Technology) was included as an internal standard to monitor loading errors.

### Measurement of Superoxide and TNF-α in the Culture Supernatant

The production of superoxide was determined by measuring the superoxide dismutase (SOD)-inhibitable reduction of tetrazolium salt, WST-1, as described previously with modifications [14]. C57BL/6 J mixed glia cultures in 96-well plates were treated with vehicle or iC3b in 100 µl of 1 × HBSS without phenol red. After the treatment, 50 µl of WST-1 (1 mM) in HBSS with and without 800 U/ml SOD was added immediately. The absorbance at 450 nm was read with a Spectra Max i3X microplate spectrophotometer (Molecular Devices, Sunnyvale, CA, USA), and the data at 30–40 min post-treatment were analyzed. The difference in absorbance observed in the presence and absence of SOD was considered to be the amount of superoxide produced, and results were expressed as the percentage of vehicle-treated control cultures.

The levels of tumor necrosis factor α (TNF-α) in the culture medium were measured with commercial ELISA kits from R&D Systems following the manufacturer’s instructions. The results were quantified using a Spectra Max i3X microplate spectrophotometer.

### Statistics

Data were presented as the mean ± SEM. Comparisons of more than two groups were performed using one-way ANOVA followed by the Bonferroni post hoc multiple comparison test. Comparisons of more than two parameters were performed by two-way ANOVA analysis followed by different post hoc multiple comparison tests. Data were analyzed using Prism (v6.00, GraphPad, San Diego, CA). *P*-values less than or equal to 0.05 were considered statistically significant.

## Results

### LPS Treatment Enhances C3 Expression in Primary Neuron/Glial Cultures

**Fig. 1:**
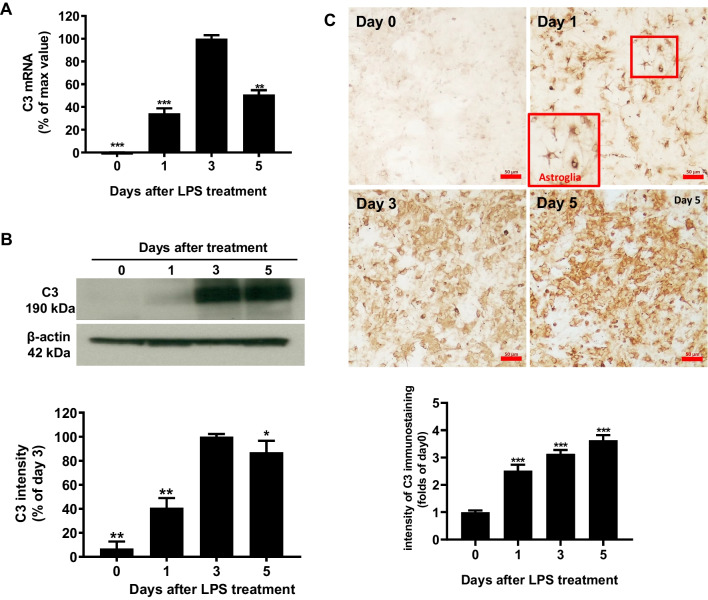
Mouse primary midbrain neuron/glial cultures were treated with LPS (15 ng/ml), and expression of C3 was detected at different time points after treatment (1, 3, 5 days). **A** C3 mRNA levels were detected by real-time PCR analysis. **B** Western blot analysis was quantified by densitometry. **C** Immunostaining pictures were performed with an antibody against C3, and quantitative values were obtained with Image J. Scale bar: 50 μm. Values are expressed as mean ± SEM from at least three independent experiments in duplicate. *p < 0.05, **p < 0.01, ***p < 0.001. Data were analyzed with one-way ANOVA followed by Dunnett’s post hoc multiple comparisons test

To study the molecular mechanism of C3 regulation in brain tissues, we used LPS-treated primary mouse neuron/glial cell cultures to determine the expression of complement C3. C57BL/6 J neuron/glial cultures were treated with LPS (15 ng/ml). The LPS treatment causes a time-dependent increase in C3 mRNA expression, and the C3 mRNA peaked at day 3 after LPS stimulation (Fig. 1A). Western blot analysis revealed significant increases in cellular levels of C3 protein comparable to that of C3 mRNA (Fig. 1B). The increase in the expression of C3 after LPS treatment was also shown in the immunocytochemical staining studies (Fig. 1C). Image J software was used to detect and quantify the gray degree values of the positive signals in the Western blot and staining. Adjustments for the background of all images had been made identically. Morphological observations suggested that the majority of the C3-positive cells are astroglia (Fig. 1C).

### The Presence of Microglia is Essential in LPS-Elicited Increase in C3 mRNA in Astroglia-Enriched Cultures

**Fig. 2:**
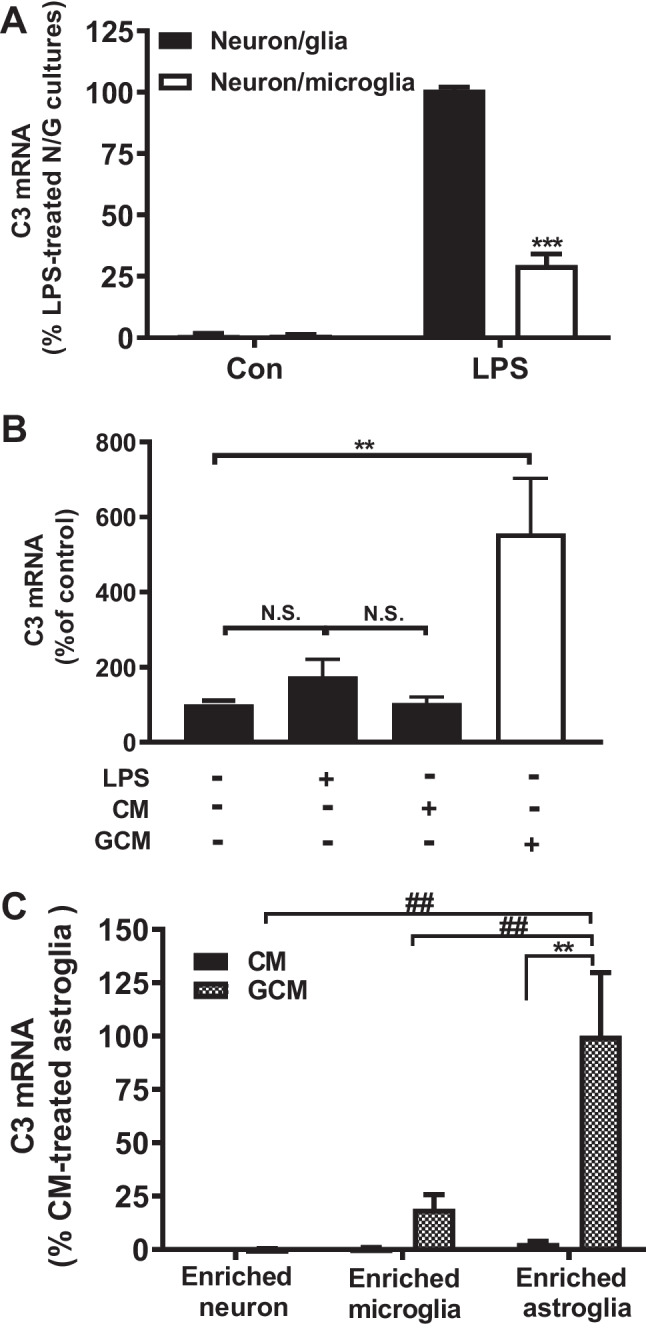
Expression levels of LPS-induced C3 mRNA in different culture conditions. **A** Primary neuron/glial and neuron-microglia (without astroglia) cultures were treated with LPS 15 ng/ml. C3 mRNA was detected on day 1 after treatment. Note that the level of C3 mRNA of neuron-glial culture, which showed the highest increase in response to LPS treatment, was set as 100%. This way showed a clearer picture between the treatment groups. **B** C57BL/6 J neuron-astroglial cultures were treated with LPS, control-conditioned medium (CM), and glia-conditioned medium (GCM). C3 mRNA was detected on day 3 after treatment. **C** Astroglia-, neuron-, and microglia-enriched cultures were treated with GCM; C3 mRNA was detected 1 day after treatment. Note that the level of C3 mRNA of enriched astroglia, which showed the highest increase in response to LPS treatment, was set as 100% to show a clearer picture indicating the differences between the treatment groups. All results are mean ± SEM from at least three independent experiments in duplicate. ** and ## *p* < 0.01, *** *p* < 0.0001. Panels **A** and **C** were analyzed with two-way ANOVA followed by Dunnett’s post hoc multiple comparisons test. Panel **B** was analyzed with one-way ANOVA followed by Dunnett’s post hoc multiple comparisons test

To further investigate the cell types expressing C3 in the neuron/glial cultures, we prepared different neuron and glia reconstituted cultures and enriched cultures. Cell number was counted before seeding to assure the numbers of microglia presented in neuron/glia and neuron/microglial cultures were comparable. Since morphological evidence suggests C3 immunostaining appears mainly in astroglia (Fig. 1C), we first performed a reconstituted neuron-microglial culture, which does not contain astroglia. LPS treatment produced much less C3 mRNA expression compared with that of neuron/glial cultures (Fig. 2A). Further mechanistic studies revealed that the presence of microglia is essential for LPS-elicited increase in C3 expression in astroglia. We found that LPS failed to induce C3 expression in neuron-astroglial co-cultures, which lack microglia (Fig. 2B). To test if proinflammatory factors released from activated microglia after LPS stimulation mediates C3 expression, we added the LPS-treated glial conditioned medium (GCM) or control conditioned medium (CM) to neuron-astroglial cultures, and assessed changes in C3 mRNA. The C3 mRNA was significantly increased in GCM-treated neuron-astroglial cultures but not in CM-treated cultures (Fig. 2B). We further treated astroglia-, microglia-, and neuron-enriched cultures with MCM to determine the major cell type in producing C3. The results indicate that astroglia are the major source of C3 in the brain tissues, while microglia and neuron expressed about 25% and 1% of astroglia C3 levels, respectively (Fig. 2C). The induction of C3 in astroglia requires the proinflammatory factors released from activated microglia during neuroinflammation.

### Continuing Release of DAMPs from Damaged/Dead Brain Cells Contributes to the Long-Lasting Expression of C3

**Fig. 3 (Figure 3 is missing, the file is in the attachment):**
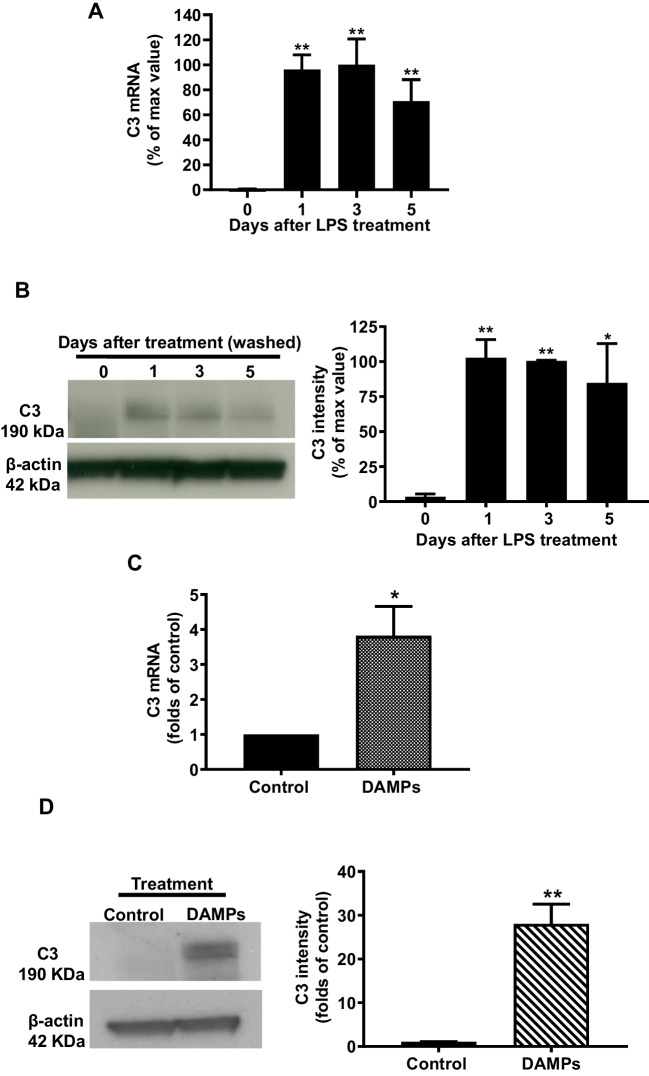
Culture supernatants were withdrawn and washed 24 h after LPS treatment and replenished with a fresh treatment medium to remove LPS and proinflammatory factors produced. At various time points after washout, the expression of **A** C3 mRNA and **B** protein was detected in neuron/glia cultures (N/G). **C** C3 mRNA in C57BL/6 J neuron/glia cultures was measured on day 3 after damage-associated molecular patterns (DAMPs) conditioned medium treatment. **D** Western blot analysis for C3 expression and quantified data in C57BL/6 J NG on day5 after DAMPs treatment. The reason for measuring mRNA and protein at different time points is to allow a sufficient period for the translation of mRNA to protein. All results are mean ± SEM from at least three independent experiments in duplicate. *p < 0.05, **p < 0.01, and *** *p* < 0.001. Panels **A** and **B** were analyzed with one-way ANOVA followed by Dunnett’s post hoc multiple comparisons test. Panels **C** and **D** were analyzed with unpaired *t*-test

A recent study shows that the presence of proinflammatory factors such as TNF-α, IL-1α, and C1q released from activated microglia triggers C3 expression in astroglia during acute neuroinflammation [27]. However, the half-lives of these released factors are fairly short. Thus, some other factors may likely be necessary for maintaining the long-lasting expression of C3 as shown in Fig. 1. To investigate how C3 expression was maintained in chronic neuroinflammation, LPS (15 ng/ml) was given once, then LPS and released inflammatory factors from activated microglia were removed by washing the neuron/glial cultures 24 h after treatment. Compared with pre-washed levels (day 1), expression of C3 mRNA and protein in washed cultures was still maintained at the same magnitudes through day 5 (Fig. 3A, B). We hypothesized that the release of endogenous molecules named DAMPs from damaged or death of cells caused by LPS-induced neuroinflammation might link with the long-lasting expression of C3. To test this possibility, the conditioned medium containing DAMPs was collected from LPS-treated neuron/glial cultures, as described in the “Materials and Methods” section. Control conditioned medium or DAMP-containing conditioned medium was added to neuron/glial cultures. Three days after treatment, C3 mRNA and protein expressions were significantly increased in DAMP-containing conditioned medium-treated cultures (Fig. 3C, D). These results suggested that LPS and the inflammatory factors released from activated microglia are necessary to induce C3 expression during acute neuroinflammation. By contrast, C3 expression in chronic neuroinflammation was sustained by the DAMPs released from damaged or dying cells.

### The Mac1-NOX2 Signaling Axis Is Critical in Mediating Long-Lasting C3 Expression

**Fig. 4:**
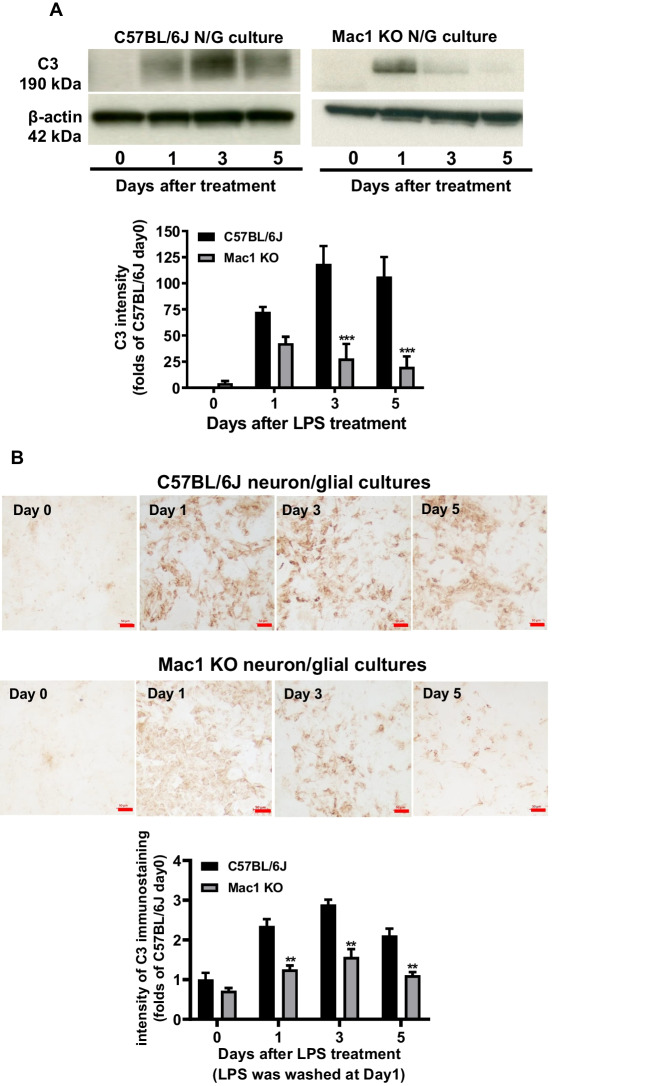
LPS and proinflammatory factors were washed out and a fresh medium was added back to the cell cultures 24 h after LPS treatment. C3 protein was determined by **A** Western blot in C57BL/6 J and Mac1-deficient neuron/glia cultures. **B** Immunocytochemical staining and quantification of C3 expression in C57BL/6 J and Mac1 KO neuron/glia cultures treated with 15 ng/ml LPS (LPS was washed out at 24 h after treatment). Scale bar: 50 μm. All results are mean ± SEM from at least three independent experiments in duplicate. **p < 0.01. Data were analyzed with two-way ANOVA followed by Sidak’s post hoc multiple comparisons test

Activation of microglial C3 receptor, Mac1 (also knowns as complement receptor 3 [CR3], CD11b/CD18 or α_M_β_2_), by DAMP molecules, such as α-synuclein, β-amyloid, and HMGB-1 released from damaged cells, has been shown to cause chronic microgliosis and sustain neuroinflammation [10, 12, 14]. To elucidate whether Mac1 is involved in C3 expression in chronic neuroinflammation, wild-type and Mac1-deficient neuron/glial cultures were treated with LPS and washed 24 h after treatment. Western blot analysis revealed that after washout, time-dependent decreases in C3 protein in Mac1 KO neuron/glia cultures were observed. Compared with levels of C57BL/6 J, 50%, 70%, and 80% decreases in C3 immunoreactivity were found at 1, 3, and 5 days, respectively (Fig. 4A). The results from immunostaining analysis showed a similar pattern of change as that of western analysis (Fig. 4B). Cell counting at the end of the experiment showed that the ratios of neuron, microglia, and astroglia remain comparable between wild-type and Mac1-deficient neuron/glial cultures, indicating the difference in LPS-elicited C3 expression was not due to the change of cell ratio.

**Fig. 5:**
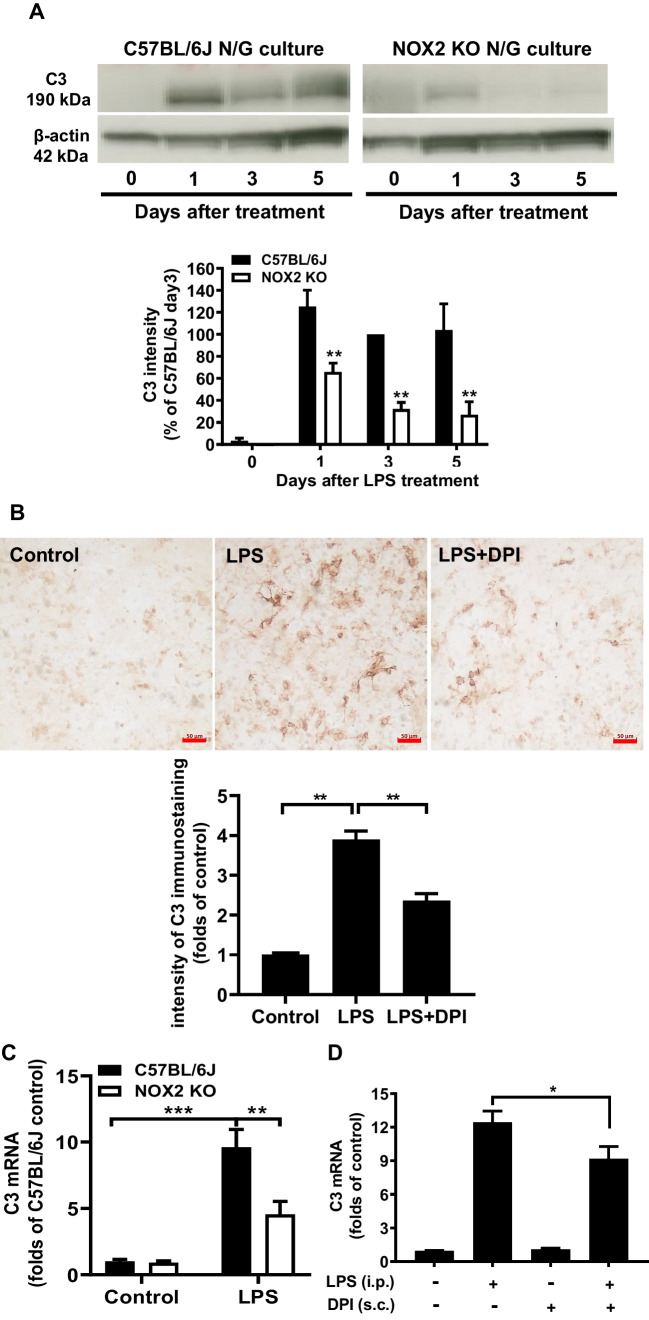
**A** C3 protein expression was detected by Western blot in C57BL/6 J and NOX2-deficient (NOX2 KO) neuron/glia cultures (N/G). Western blot was qualified by densitometry. **B** Immunostaining of C3 expression of C57BL/6 J neuron/glial cultures treated with LPS and LPS plus DPI (10^−13^ M), a NOX2 inhibitor. Scale bar: 50 μm. **C** C57BL/6 J and NOX-2 KO mice were treated with LPS (3.5 mg/kg i.p.). Brain C3 mRNA in C57BL/6 J and NOX2-deficient mice 24 h after LPS injection. **D** Brain C3 mRNA levels in LPS and LPS + DPI injected C57BL/6 J mice were measured 24 h after LPS injection. We pre-treated C57BL/6 J mice with saline or 10 ng/kg DPI for 4 days by subcutaneous injection (3 times per day) before the single LPS administration, then continuously post-treated at 6 and 12 h after LPS injection. All brain samples were collected 24 h after LPS to determine the brain C3 mRNA level. There are at least three mice in each group. All results are mean ± SEM from at least three independent experiments in duplicate. *P < 0.05, **P < 0.01. Panels **A** and **C** were analyzed with two-way ANOVA followed by Sidak’s post hoc multiple comparisons test. Panels **B** and **D** were analyzed with one-way ANOVA followed by Dunnett’s post hoc multiple comparisons test

Reports from our laboratory and others have provided strong evidence indicating that microglial NADPH oxidase2 (NOX2) is critical in mediating toxin-elicited microglial activation leading to neuroinflammation [20, 28-30]. We demonstrated that LPS-induced microglial activation and subsequent neuronal death were greatly reduced in NOX2-deficient conditions in vivo and in vitro [20, 28]. Since it is well-documented that NOX2 is one of the major downstream effectors of Mac1 [10], we examined the role of NOX2 in C3 regulation. Upon the activation of the Mac1 by C3 or DAMPs, PI3 kinase was activated to trigger the phosphorylation of cytosolic subunits of NOX2, which in turn translocate and bind to membrane subunits to generate superoxide and related ROS [31]. Wild-type and Mac1-deficient neuron/glial cultures were treated with LPS and washed 24 h after treatment. The essential role of NOX2 in the regulation of C3 expression was illustrated in evidence showing that LPS-elicited increase in C3 protein expression was significantly decreased in NOX2-deficient neuron/glial cultures (Fig. 5A). Moreover, the immunocytochemical analysis showed that post-treatment with DPI, a NOX2 inhibitor, LPS-induced increase in C3 expression was decreased in wild-type cultures (Fig. 5B). To determine whether NOX2 regulates brain C3 expression in vivo, we used an inflammation-mediated neurodegeneration mouse model through a single systemic injection of LPS. Brain C3 mRNA in NOX2 KO mice was 50% lower than that of in wild-type control 24 h after LPS injection (Fig. 5C). Similar results were obtained in LPS-injected mice, which were treated with the NOX2 inhibitor DPI (Fig. 5D). Taken together, strong evidence indicates that the C3- Mac1-NOX2 signal axis plays a critical role in maintaining long-lasting C3 expression.

### C3 Increases Superoxide Production and Elevates LPS-Induced Oxidative Stress

**Fig. 6:**
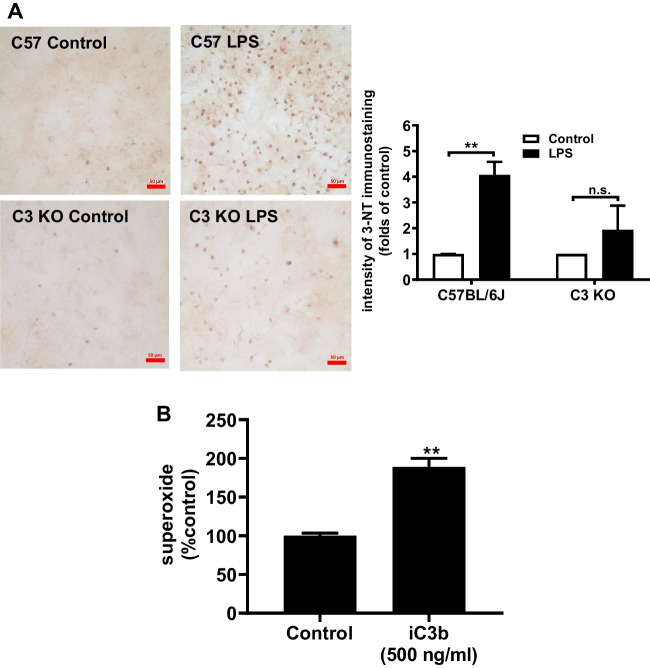
C3 increases the production of superoxide-mediated oxidative stress and enhances neuron damage through the activation of the Mac1-NOX2 axis. **A** Immunocytochemical staining of 3-nitrotyrosine (3-NT), an oxidative stress marker, in C57BL/6 J, and C3 KO neuron/glia cultures at 7 days after LPS treatment. All results are mean ± SEM from at least three independent experiments in duplicate. *p < 0.05 and **p < 0.01. **B** Superoxide level was detected in endotoxin-free iC3b (500 ng/ml) treated C57BL/6 J mixed glial cultures. Panel **A** was analyzed with two-way ANOVA flowed by Sidak’s post hoc multiple comparisons test. Panel **B** was analyzed with an unpaired *t*-test

Our previous study demonstrated that the elevation of oxidative stress partly resulted from the Mac1-NOX2 axis-produced superoxide/ROS is a pivotal feature of neuroinflammation and neurodegeneration [32-35]. To further elucidate the role of C3 in chronic neuroinflammation, we compared the oxidative stress in wild-type and C3-deficient neuron/glial cultures using an antibody against 3-NT, which is a marker for oxidative stress. The number of 3-NT-positive cells was significantly increased in wild-type cultures than that in C3-deficient neuron/glial culture 7 days after 15 ng/ml LPS treatment, indicating that the oxidative stress was reduced without C3 expression (Fig. 6A). We also determined the superoxide production in endotoxin-free iC3b (an active metabolite of C3)-treated wild-type mixed glial cultures. Compared with the control group, the iC3b treatment significantly increased superoxide production in mixed glial cultures (Fig. 6B).

### The Absence of C3 Prevents LPS-Elicited Progressive Neurodegeneration

**Fig. 7:**
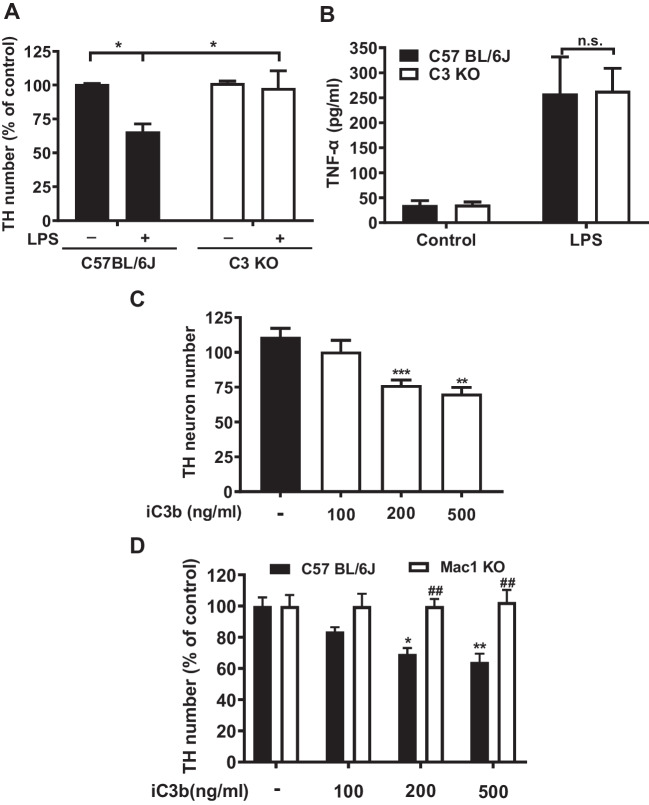
Lack of C3 prevents LPS-elicited progressive neurodegeneration. To achieve a greater degree of TH neuronal loss, a higher concentration of LPS (20 ng/ml) was used for both panels **A** and **B**. **A** Significant loss of TH neuron number in C57BL/6 J, but not in C3-deficient neuron/glial cultures 7 days after LPS (20 ng/ml) treatment. **B** LPS produced similar increases in supernatant TNF-α levels in both C57BL/6 J and C3-deficient neuron/glia cultures 3 h after treatment. **C** Dose-dependent loss of TH neurons in iC3b-treated in C57BL/6 J neuron/glial cultures 7 days after treatment. **D** Graded loss of TH neurons in C57BL/6 J, but not in Mac1-deficient neuron/glial cultures 7 days after various concentrations of iC3b treatment. All results are mean ± SEM from at least three independent experiments in duplicates. *p < 0.05, ** and ## *p* < 0.01. Panels **A**, **B**, and **D** were analyzed with two-way ANOVA followed by Sidak’s post hoc multiple comparisons test. Panel **C** was analyzed with one-way ANOVA followed by Dunnett’s post hoc multiple comparisons test

To further understand how C3 participates in the pathogenesis of neurodegeneration, we tested the hypothesis that upregulated C3 could over-activate microglia to trigger neuron loss in chronic neuroinflammation. Wild-type and C3-deficient neuron/glial cultures were treated with LPS, and the dopaminergic neuron numbers were counted after staining with its marker TH 7 days after treatment. There is about 30% neuronal loss in wild-type neuron/glial cultures; by contrast, there is no significant neuronal loss in C3-deficient-neuron/glial cultures (Fig. 7A). We had previously reported that activated microglia is the major source of production TNF-α [24]. LPS-elicited increases in supernatant TNF-α levels were similar between C3 KO and wild-type neuron/glia cultures (Fig. 7B). These results suggest that in the absence of C3, LPS still can produce acute inflammation and microglia activation during the first few hours after treatment and provides clear evidence indicating that C3 is needed for maintaining chronic neuroinflammation and triggering neurodegeneration.

To directly determine whether C3 can cause neuron damage, different amounts of iC3b, the active form of C3, were added to wild-type neuron/glial cultures. iC3b dose-dependently caused loss of dopaminergic neurons in wild-type neuron/glial cultures (Fig. 7C). We also used the wild-type and Mac1-deficient neuron/glial cultures and observed neurodegeneration in treated cultures. By contrast, iC3b did not produce and loss of dopaminergic neurons in Mac1-deficient neuron/glial cultures (Fig. 7D), suggesting that iC3b binds to the Mac1 receptor to activate microglia and subsequently causes damage to dopaminergic neurons.

### A Systemic LPS Injection Caused a Sustained Increase in C3 Expression and Oxidative Stress in the Brain

**Fig. 8:**
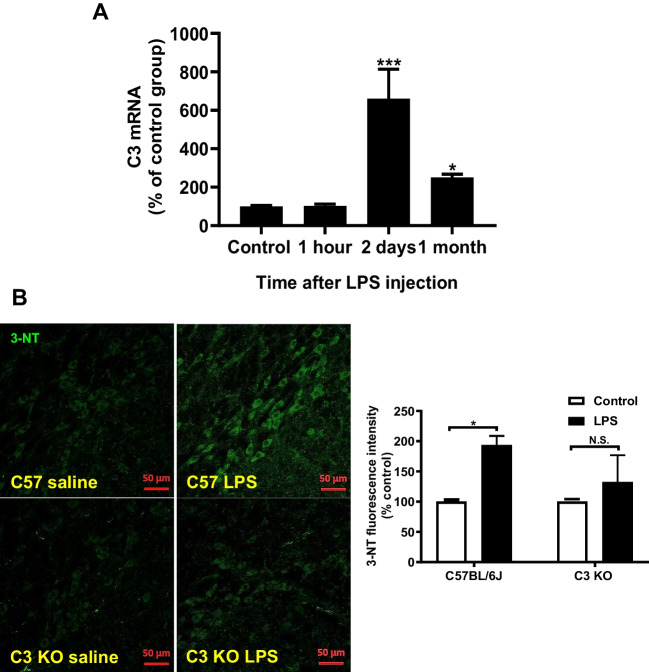
**A** C3 mRNA in LPS-injected wild-type mouse brains at 1 h, 2 days, and 1 month after the injection. *N* = 5. **B** Representative pictures of immunofluorescence staining of 3-NT (green color) in the substantial nigra region of LPS-injected C57BL/6 J and C3-deficient mice 6 months after injection. Note the immunofluorescence staining of 3-NT was mainly observed in the substantial nigra compact region where the majority of cell type was TH-positive dopamine-containing neurons. There are three mice in each group. All results are mean ± SEM from at least three independent experiments in duplicate. *p < 0.05, **p < 0.01, and *** *p* < 0.001. Panel **A** was analyzed with an unpaired *t*-test. Panel **B** was analyzed with two-way ANOVA flowed by Sidak’s post hoc multiple comparisons test

An in vivo experiment was performed to confirm that the abovementioned in vitro results were obtained from various neuron/glial cultures. RT-PCR analysis shows the increase of brain C3 mRNA levels after mice received a single injection of LPS (3.5 mg/kg,i.p.). C3 mRNA peaked at day 2 after injection and still sustained at about 250% above the control level 1 month later (Fig. 8A). Furthermore, we also determined whether the increase in complement C3 expression is associated with neuronal oxidative stress observed in LPS-injected mice during the late chronic neuroinflammatory stage. The nitrosative stress and oxidative stress marker 3-NT was measured by immunofluorescence staining in the substantial nigra in both wild-type and C3-deficient mice 6 months after LPS injection. The results showed that the LPS-elicited increase in brain 3-NT immunoreactivity in C57BL/6 J mice was greatly reduced in C3-deficient mice (Fig. 8B).

## Discussion

**Fig. 9:**
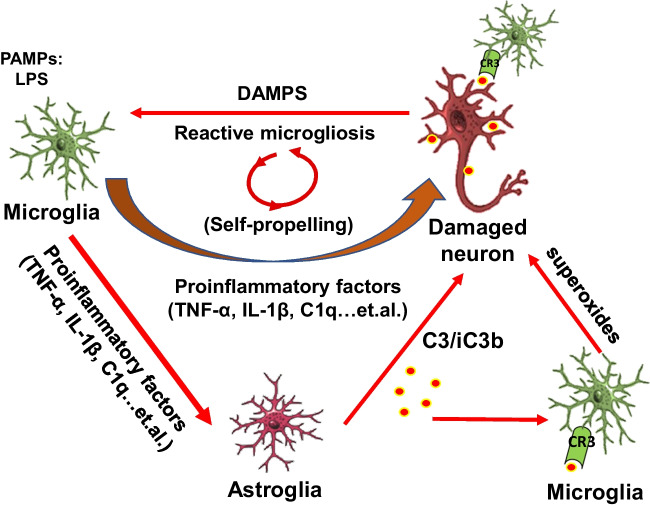
Schematic drawing showing how the possible interactions between neurons, astroglia, and microglia regulate the C3 expression and neurotoxic roles of C3 in maintaining the progression of chronic neuroinflammation and leading to neurodegeneration. During the acute phase, microglia activated by pathogen-associated molecular patterns (PAMPs), like LPS, can produce multiple proinflammatory factors to combat invading microorganisms but also can damage surrounding neurons. Proinflammatory factors released from activated microglia can also trigger astroglia to have delayed release of immune factors, including complement C3 and iC3b to label the damaged neurons during the chronic phase of neuroinflammation. The attached C3 can attract microglia to further damage neurons. Furthermore, C3 from astroglia and DAMPs from the damaged neurons or other cells can trigger the activation of the Mac1-NOX2 axis in microglia to further produce superoxides to cause oxidative stress and maintain the reactive microgliosis. During this chronic phase, microglia cease to produce proinflammatory cytokines but continue to enhance the production of C3, superoxide, and other delayed immune factors. Thus, sustaining activated microglia, astroglia, and damaged neurons can result in a self-propelling circle to maintain chronic inflammation and subsequent neurodegeneration

The present study provides additional evidence illustrating the critical role of microglia in regulating the expression of C3 in astroglia. The dual function of C3 in affecting neuronal function and reactive microgliosis is summarized in Fig. 9. Multiple proinflammatory factors released from over-activated microglia resulting from LPS stimulation can produce damage to neurons and also trigger the activation of astroglia [24]. Activated astroglia can produce C3 and iC3b to label the damaged neurons [27]. On the one hand, the C3 attached to neurons can attract microglia to remove damaged neurons through phagocytosis. On the other hand, C3 released from astroglia and DAMPs from the damaged neurons can trigger the reactivation of microglia through the activation of the Mac1-NOX2 axis in microglia. Substantial evidence supports the formation of a self-propelling circle among activated microglia, astroglia, and damaged neurons to sustain the long-lasting C3 expression, which in turn participates in maintaining chronic neuroinflammation and driving subsequent neurodegeneration (Fig. 9).

### Microglia Are Essential in LPS-Elicited Increase in C3 mRNA in Cultured Astroglia

During acute inflammation, blood macromolecules like complements cannot freely penetrate the BBB. Thus, complement components produced locally in the brain are critical immune responses to infections and traumatic brain injury. Our in vitro studies showed that C3 mRNA significantly diminished in neuron-microglia cultures (lacking astroglia) after LPS stimulation (Fig. 2A) which is consistent with previous studies indicating that the majority of complement components found in the brain are produced from astroglia [27, 36, 37]. However, the mechanism regulating the production of C3 in astroglia is not clear. LPS was previously thought to directly upregulate the expression of complement C3 in primary astroglial cultures [5, 6]. By contrast, we found that LPS failed to directly activate highly enriched astroglial cultures in our study (Fig. 2B). Additional evidence indicates that proinflammatory factors released from LPS-activated microglia are necessary to upregulate C3 in astroglia. Our finding is consistent with previous reports indicating failure of LPS in directly activating astroglia, likely due to the lack of functional TLR-4 receptors on the astroglial membrane [38, 39].

### DAMPs Released from Damaged Neurons Mediate the Long-Lasting Expression of C3

Besides the robust increase in C3 level during the acute neuroinflammation stage, the long-lasting expression of this complement has been well-described in numerous chronic neurodegenerative disorders. Increased complement C3 was found in human cerebrospinal fluid and different brain regions in Parkinson’s disease, Alzheimer’s disease (AD), and multiple system atrophy [9, 36, 37, 40-43]. Increased C3 promoted neuronal tau pathology and neurodegeneration in an AD mouse model [40, 44-46]. Furthermore, inhibition or deficiency in complement C3 showed neuroprotective effects in a cerebral ischemia model [47, 48]. However, the regulation of C3 expression in the chronic neuroinflammation stage is still not fully understood. Our finding indicated that DAMPs released from damaged/dead neurons could sustain the complement C3 expression in chronic neuroinflammation. C3 mRNA and protein are maintained at high levels even after the removal of culture supernatants (containing LPS and microglia released proinflammatory factors) in neuron/glial cultures. Moreover, the addition of DAMPs collected from damaged neurons to neuron/glial cultures increased the expression of C3 (Fig. 3C, D). It is interesting to note that DAMPs from damaged/dead brain cells, including ATP, S100, α-synuclein, β-amyloid, and HMGB-1, are known to cause reactive microgliosis by binding to microglial complement C3 receptor (Mac1). Previous studies from our and other groups showed that DAMPs like HMGB-1 are critical in inducing C3 expression and neurodegeneration [10, 49, 50]. LPS-elicited increase in the expression of C3 was greatly reduced in Mac1-deficient neuron/glial cultures (Fig. 4). Based on these findings, we conclude that DAMPs from damaged/dead neurons act on microglia Mac1 receptors to cause reactive astrogliosis and maintain C3 expression. This neuron/glial crosstalk is responsible for maintaining the self-perpetuating reactive microgliosis/astrogliosis required in chronic neuroinflammation and subsequent neurodegeneration.

### Critical Roles of Microglial Mac1-NOX2 Signaling in the Expression of C3

Recent studies have indicated microglial NOX2 is an important downstream effector of Mac1 signaling [51]. NOX2 is composed of cytosolic subunits (p47^***phox***^, p67^*phox*^, p40^*phox*^, and the small Rho GTPase, Rac1, or Rac2) and membrane-bound subunits p22 ^*phox*^ and gp91 ^*phox*^ [18]. Upon stimulation, cytosolic subunits of NOX2 translocate and bind to the membrane subunits to assemble the catalytically active form of NOX2 that produces ROS, such as extracellular superoxide and other free radicals [10, 49]. Previous studies demonstrated that neuronal DAMPs bind to Mac1 to trigger superoxide production through NOX2 activation [14, 52, 53]. Our results showed that either genetic ablation of NOX2 or pharmacologic inhibition by a NOX2 inhibitor DPI significantly reduced C3 expression both in vitro and in vivo (Fig. 5). These results suggest that neuronal DAMPs stimulate microglial ROS production through the Mac1-NOX2 axis. ROS could serve as a messenger to activate signaling molecules like NF-κB or MAP kinases to maintain the C3 expression in chronic neuroinflammation [54].

### Roles of C3 in the Pathogenesis of Chronic Inflammation-Related Neurodegeneration

Strong evidence indicates that astroglia plays both neuroprotective and neurotrophic roles by providing neurotrophic factors, buffering the toxic substances, and promoting the formation and function of synapses under normal physiological conditions [55, 56]. However, recent studies reported that a subset of reactive astroglia, termed A1, is induced by activated microglia in neuroinflammation conditions [27, 57-59]. The complement C3 is highly upregulated in A1 astroglia. A1 astroglia are present in the brains of most major neurodegenerative diseases and are closely associated with activated microglia. A1 astroglia fail to promote neuronal survival, outgrowth, synaptogenesis, and phagocytosis and may induce the death of neurons and oligodendrocytes [27]. Blockade of A1 astroglia formation is neuroprotective in neuroinflammatory conditions [57]. Our results are consistent with these findings and further provide the underlying molecular mechanisms of how C3 is regulated in the A1 astroglia.

We have addressed a critical question as to how C3 mediates the LPS-elicited progressive neurodegeneration in this study. Our results demonstrate the presence of C3 is critical in LPS-elicited progressive neurodegeneration. LPS-induced oxidative stress was significantly decreased in C3 KO neuron/glial cultures or the brain of LPS-injected C3KO mice (Fig. 6 and Fig. 8). The LPS-induced neurotoxicity was greatly reduced in C3 KO neuron/glial cultures. Further study reveals that C3-mediated neuronal toxicity is tightly regulated by the Mac1-NOX2 axis (Fig. 7). Together, these results provide strong evidence supporting the critical role of C3 in sustaining chronic neuroinflammation and participating in progressive neurodegeneration (Fig. 9).

In summary, this study demonstrated that proinflammatory factors released from activated microglia damaged neurons and triggered astroglia activation (reactive astrogliosis) to produce C3. Strong evidence further shows that C3 is one of the critical proinflammatory factors mediating and sustaining chronic neuroinflammation. The interaction between C3 and the Mac1-NOX axis not only unveils the regulation of C3 in chronic neuroinflammation but provides a potential therapeutic target for developing new interventions for neurodegenerative diseases by inhibiting the C3 production and A1 neurotoxic astroglia formation.

## Data Availability

The data used and/or analyzed during the current study are available from the corresponding author on reasonable request.

## References

[1] Korol SA (1977). Complement system (Systema komplementu). Fiziol Zh.

[2] Inoue K (1976). Complement activation and biological activities. Nihon Saikingaku Zasshi.

[3] Fearon DT, Ruddy S, Knostman JD, Carpenter CB, Austen KF (1974). The functional significance of complement. Adv Nephrol Necker Hosp.

[4] Veerhuis R, Nielsen HM, Tenner AJ (2011). Complement in the brain. Mol Immunol.

[5] Stephan AH, Barres BA, Stevens B (2012). The complement system: an unexpected role in synaptic pruning during development and disease. Annu Rev Neurosci.

[6] Baskin DS, Hosobuchi Y, Loh HH, Lee NM (1984). Dynorphin(1–13) improves survival in cats with focal cerebral ischaemia. Nature.

[7] Crehan H, Hardy J, Pocock J (2012) Microglia, Alzheimer's disease, and complement. Int J Alzheimers Dis 2012:983640. 10.1155/2012/983640 10.1155/2012/983640 PMC3432348 22957298

[8] Hong S, Beja-Glasser VF, Nfonoyim BM, Frouin A, Li S, Ramakrishnan S, Merry KM, Shi Q, Rosenthal A, Barres BA, Lemere CA, Selkoe DJ, Stevens B (2016). Complement and microglia mediate early synapse loss in Alzheimer mouse models. Science.

[9] Wang Y, Hancock AM, Bradner J, Chung KA, Quinn JF, Peskind ER, Galasko D, Jankovic J, Zabetian CP, Kim HM, Leverenz JB, Montine TJ, Ginghina C, Edwards KL, Snapinn KW, Goldstein DS, Shi M, Zhang J (2011). Complement 3 and factor h in human cerebrospinal fluid in Parkinson's disease, Alzheimer's disease, and multiple-system atrophy. Am J Pathol.

[10] Chen S-H, Oyarzabal EA, Hong J-S (2016). Critical role of the Mac1/NOX2 pathway in mediating reactive microgliosis-generated chronic neuroinflammation and progressive neurodegeneration. Curr Opin Pharmacol.

[11] Hoogland ICM, Westhoff D, Engelen-Lee J-Y, Melief J, Valls Serón M, Houben-Weerts JHMP, Huitinga I, van Westerloo DJ, van der Poll T, van Gool WA, van de Beek D (2018). Microglial activation after systemic stimulation with lipopolysaccharide and Escherichia coli. Front Cell Neurosci.

[12] Hou L, Wang K, Zhang C, Sun F, Che Y, Zhao X, Zhang D, Li H, Wang Q (2018). Complement receptor 3 mediates NADPH oxidase activation and dopaminergic neurodegeneration through a Src-Erk-dependent pathway. Redox Biol.

[13] Kim KI, Chung YC, Jin BK (2018). Norfluoxetine prevents degeneration of dopamine neurons by inhibiting microglia-derived oxidative stress in an MPTP mouse model of Parkinson's disease. Mediators Inflamm.

[14] Gao H-M, Zhou H, Zhang F, Wilson BC, Kam W, Hong J-S (2011). HMGB1 acts on microglia Mac1 to mediate chronic neuroinflammation that drives progressive neurodegeneration. J Neurosci.

[15] Umeno A, Biju V, Yoshida Y (2017). In vivo ROS production and use of oxidative stress-derived biomarkers to detect the onset of diseases such as Alzheimer's disease, Parkinson's disease, and diabetes. Free Radical Res.

[16] Dias V, Junn E, Mouradian MM (2013). The role of oxidative stress in Parkinson's disease. J Parkinsons Dis.

[17] Loffredo L, Ettorre E, Zicari AM, Inghilleri M, Nocella C, Perri L, Spalice A, Fossati C, De Lucia MC, Pigozzi F, Cacciafesta M, Violi F, Carnevale R (2020). Oxidative stress and gut-derived lipopolysaccharides in neurodegenerative disease: role of NOX2. Oxid Med Cell Longev.

[18] Ma MW, Wang J, Zhang Q, Wang R, Dhandapani KM, Vadlamudi RK, Brann DW (2017). NADPH oxidase in brain injury and neurodegenerative disorders. Mol Neurodegener.

[19] Douda DN, Khan MA, Grasemann H, Palaniyar N (2015). SK3 channel and mitochondrial ROS mediate NADPH oxidase-independent NETosis induced by calcium influx. Proc Natl Acad Sci USA.

[20] Qin L, Liu Y, Wang T, Wei SJ, Block ML, Wilson B, Liu B, Hong JS (2004). NADPH oxidase mediates lipopolysaccharide-induced neurotoxicity and proinflammatory gene expression in activated microglia. J Biol Chem.

[21] Wang Q, Qian L, Chen SH, Chu CH, Wilson B, Oyarzabal E, Ali S, Robinson B, Rao D, Hong JS (2015). Post-treatment with an ultra-low dose of NADPH oxidase inhibitor diphenyleneiodonium attenuates disease progression in multiple Parkinson's disease models. Brain : J Neurol.

[22] Wang Q, Chu C-H, Oyarzabal E, Jiang L, Chen S-H, Wilson B, Qian L, Hong J-S (2014). Subpicomolar diphenyleneiodonium inhibits microglial NADPH oxidase with high specificity and shows great potential as a therapeutic agent for neurodegenerative diseases. Glia.

[23] Qin L, Wu X, Block ML, Liu Y, Breese GR, Hong J-S, Knapp DJ, Crews FT (2007). Systemic LPS causes chronic neuroinflammation and progressive neurodegeneration. Glia.

[24] Chen S-H, Oyarzabal EA, Sung Y-F, Chu C-H, Wang Q, Chen S-L, Lu R-B, Hong J-S (2015). Microglial regulation of immunological and neuroprotective functions of astroglia. Glia.

[25] Wang Q, Chu CH, Oyarzabal E, Jiang L, Chen SH, Wilson B, Qian L, Hong JS (2014). Subpicomolar diphenyleneiodonium inhibits microglial NADPH oxidase with high specificity and shows great potential as a therapeutic agent for neurodegenerative diseases. Glia.

[26] Chen SH, Oyarzabal EA, Hong JS (2013). Preparation of rodent primary cultures for neuron-glia, mixed glia, enriched microglia, and reconstituted cultures with microglia. Methods Mol Biol (Clifton, NJ).

[27] Liddelow SA, Guttenplan KA, Clarke LE, Bennett FC, Bohlen CJ, Schirmer L, Bennett ML, Munch AE, Chung WS, Peterson TC, Wilton DK, Frouin A, Napier BA, Panicker N, Kumar M, Buckwalter MS, Rowitch DH, Dawson VL, Dawson TM, Stevens B, Barres BA (2017). Neurotoxic reactive astrocytes are induced by activated microglia. Nature.

[28] Qin L, Liu Y, Hong JS, Crews FT (2013). NADPH oxidase and aging drive microglial activation, oxidative stress, and dopaminergic neurodegeneration following systemic LPS administration. Glia.

[29] Choi SH, Aid S, Kim HW, Jackson SH, Bosetti F (2012). Inhibition of NADPH oxidase promotes alternative and anti-inflammatory microglial activation during neuroinflammation. J Neurochem.

[30] Huang WY, Lin S, Chen HY, Chen YP, Chen TY, Hsu KS, Wu HM (2018). NADPH oxidases as potential pharmacological targets against increased seizure susceptibility after systemic inflammation. J Neuroinflammation.

[31] Check J, Byrd CL, Menio J, Rippe RA, Hines IN, Wheeler MD (2010). Src kinase participates in LPS-induced activation of NADPH oxidase. Mol Immunol.

[32] Gao Y, Tu D, Yang R, Chu C-H, Hong J-S, Gao H-M (2020). Through reducing ROS production, IL-10 suppresses caspase-1-dependent IL-1β maturation, thereby preventing chronic neuroinflammation and neurodegeneration. Int J Mol Sci.

[33] Song S, Wang Q, Jiang L, Oyarzabal E, Riddick NV, Wilson B, Moy SS, Shih YI, Hong JS (2019). Noradrenergic dysfunction accelerates LPS-elicited inflammation-related ascending sequential neurodegeneration and deficits in non-motor/motor functions. Brain Behav Immun.

[34] Qin L, Liu Y, Hong JS, Crews FT (2013). NADPH oxidase and aging drive microglial activation, oxidative stress, and dopaminergic neurodegeneration following systemic LPS administration. Glia.

[35] Gao HM, Zhou H, Hong JS (2012). NADPH oxidases: novel therapeutic targets for neurodegenerative diseases. Trends Pharmacol Sci.

[36] Hernandez-Encinas E, Aguilar-Morante D, Morales-Garcia JA, Gine E, Sanz-SanCristobal M, Santos A, Perez-Castillo A (2016). Complement component 3 (C3) expression in the hippocampus after excitotoxic injury: role of C/EBPbeta. J Neuroinflammation.

[37] Lian H, Litvinchuk A, Chiang AC, Aithmitti N, Jankowsky JL, Zheng H (2016). Astrocyte-microglia cross talk through complement activation modulates amyloid pathology in mouse models of Alzheimer's disease. J Neurosci.

[38] Jiang H, Wang Y, Liang X, Xing X, Xu X, Zhou C (2018). Toll-like receptor 4 knockdown attenuates brain damage and neuroinflammation after traumatic brain injury via inhibiting neuronal autophagy and astrocyte activation. Cell Mol Neurobiol.

[39] Rahimifard M, Maqbool F, Moeini-Nodeh S, Niaz K, Abdollahi M, Braidy N, Nabavi SM, Nabavi SF (2017). Targeting the TLR4 signaling pathway by polyphenols: a novel therapeutic strategy for neuroinflammation. Ageing Res Rev.

[40] Wu T, Dejanovic B, Gandham VD, Gogineni A, Edmonds R, Schauer S, Srinivasan K, Huntley MA, Wang Y, Wang T-M, Hedehus M, Barck KH, Stark M, Ngu H, Foreman O, Meilandt WJ, Elstrott J, Chang MC, Hansen DV, Carano RAD, Sheng M, Hanson JE (2019). Complement C3 is activated in human AD brain and is required for neurodegeneration in mouse models of amyloidosis and tauopathy. Cell Rep.

[41] Bharara S, Sorscher EJ, Gillespie GY, Lindsey JR, Hong JS, Curlee KV, Allan PW, Gadi VK, Alexander SA, Secrist JA, Parker WB, Waud WR (2005). Antibiotic-mediated chemoprotection enhances adaptation of E. coli PNP for herpes simplex virus-based glioma therapy. Hum Gene Ther.

[42] Napier BA, Brubaker SW, Sweeney TE, Monette P, Rothmeier GH, Gertsvolf NA, Puschnik A, Carette JE, Khatri P, Monack DM (2016). Complement pathway amplifies caspase-11-dependent cell death and endotoxin-induced sepsis severity. J Exp Med.

[43] Bodea L-G, Wang Y, Linnartz-Gerlach B, Kopatz J, Sinkkonen L, Musgrove R, Kaoma T, Muller A, Vallar L, Di Monte DA, Balling R, Neumann H (2014). Neurodegeneration by activation of the microglial complement-phagosome pathway. J Neurosci.

[44] Litvinchuk A, Wan Y-W, Swartzlander DB, Chen F, Cole A, Propson NE, Wang Q, Zhang B, Liu Z, Zheng H (2018). Complement C3aR inactivation attenuates tau pathology and reverses an immune network deregulated in tauopathy models and Alzheimer's Disease. Neuron.

[45] Vogels T, Murgoci A-N, Hromádka T (2019). Intersection of pathological tau and microglia at the synapse. Acta Neuropathol Commun.

[46] Davies C, Spires-Jones TL (2018). Complementing Tau: New Data Show that the Complement System Is Involved in Degeneration in Tauopathies. Neuron.

[47] Alawieh A, Langley E F, Tomlinson S (2018) Targeted complement inhibition salvages stressed neurons and inhibits neuroinflammation after stroke in mice. Sci Transl Med 10(441). 10.1126/scitranslmed.aao6459 10.1126/scitranslmed.aao6459 PMC6689196 29769288

[48] Lai W, Xie X, Zhang X, Wang Y, Chu K, Brown J, Chen L, Hong G (2018). Inhibition of complement drives increase in early growth response proteins and neuroprotection mediated by salidroside after cerebral ischemia. Inflammation.

[49] Kumar A, Barrett JP, Alvarez-Croda DM, Stoica BA, Faden AI, Loane DJ (2016). NOX2 drives M1-like microglial/macrophage activation and neurodegeneration following experimental traumatic brain injury. Brain Behav Immun.

[50] Gao HM, Zhou H, Zhang F, Wilson BC, Kam W, Hong JS (2011). HMGB1 acts on microglia Mac1 to mediate chronic neuroinflammation that drives progressive neurodegeneration. J Neurosc : Off J Soc Neurosci.

[51] Zhang J, Malik A, Choi HB, Ko RWY, Dissing-Olesen L, MacVicar BA (2014). Microglial CR3 activation triggers long-term synaptic depression in the hippocampus via NADPH oxidase. Neuron.

[52] Hu X, Zhang D, Pang H, Caudle WM, Li Y, Gao H, Liu Y, Qian L, Wilson B, Di Monte DA, Ali SF, Zhang J, Block ML, Hong JS (2008). Macrophage antigen complex-1 mediates reactive microgliosis and progressive dopaminergic neurodegeneration in the MPTP model of Parkinson's disease. J Immunol.

[53] Zhang D, Hu X, Qian L, Chen SH, Zhou H, Wilson B, Miller DS, Hong JS (2011). Microglial MAC1 receptor and PI3K are essential in mediating β-amyloid peptide-induced microglial activation and subsequent neurotoxicity. J Neuroinflammation.

[54] Zhang J, Wang X, Vikash V, Ye Q, Wu D, Liu Y, Dong W (2016). ROS and ROS-mediated cellular signaling. Oxid Med Cell Longev.

[55] Verkhratsky A, Nedergaard M (2018). Physiology of astroglia. Physiol Rev.

[56] Verkhratsky A, Ho MS, Vardjan N, Zorec R, Parpura V (2019). General pathophysiology of astroglia. Adv Exp Med Biol.

[57] Yun SP, Kam TI, Panicker N, Kim S, Oh Y, Park JS, Kwon SH, Park YJ, Karuppagounder SS, Park H, Kim S, Oh N, Kim NA, Lee S, Brahmachari S, Mao X, Lee JH, Kumar M, An D, Kang SU, Lee Y, Lee KC, Na DH, Kim D, Lee SH, Roschke VV, Liddelow SA, Mari Z, Barres BA, Dawson VL, Lee S, Dawson TM, Ko HS (2018). Block of A1 astrocyte conversion by microglia is neuroprotective in models of Parkinson's disease. Nat Med.

[58] Joshi AU, Minhas PS, Liddelow SA, Haileselassie B, Andreasson KI, Dorn GW, Mochly-Rosen D (2019). Fragmented mitochondria released from microglia trigger A1 astrocytic response and propagate inflammatory neurodegeneration. Nat Neurosci.

[59] Hinkle JT, Dawson VL, Dawson TM (2019). The A1 astrocyte paradigm: new avenues for pharmacological intervention in neurodegeneration. Mov Disord : Off J Mov Disord Soc.

